# Engineering global and local signal generators for probing temporal and spatial cellular signaling dynamics

**DOI:** 10.3389/fbioe.2023.1239026

**Published:** 2023-09-14

**Authors:** Haowen Yang, Jurjen Tel

**Affiliations:** ^1^ Laboratory of Immunoengineering, Department of Biomedical Engineering, Eindhoven University of Technology, Eindhoven, Netherlands; ^2^ Institute for Complex Molecular Systems, Eindhoven University of Technology, Eindhoven, Netherlands

**Keywords:** signal generator, microfluidics, signaling dynamics, single cells, cellular communication

## Abstract

Cells constantly encounter a wide range of environmental signals and rely on their signaling pathways to initiate reliable responses. Understanding the underlying signaling mechanisms and cellular behaviors requires signal generators capable of providing diverse input signals to deliver to cell systems. Current research efforts are primarily focused on exploring cellular responses to global or local signals, which enable us to understand cellular signaling and behavior in distinct dimensions. This review presents recent advancements in global and local signal generators, highlighting their applications in studying temporal and spatial signaling activity. Global signals can be generated using microfluidic or photochemical approaches. Local signal sources can be created using living or artificial cells in combination with different control methods. We also address the strengths and limitations of each signal generator type, discussing challenges and potential extensions for future research. These approaches are expected to continue to facilitate on-going research to discover novel and intriguing cellular signaling mechanisms.

## Introduction

Cells possess the remarkable capability to perceive and respond to a wide array of time-varying signals from their environment. This ability stems from a diverse functional repertoire of genes, proteins, and metabolites that interact in response to various external physical cues, such as matrix stiffness (Discher et al., 2005) and fluid shear stress (Chen et al., 2019), as well as biochemical cues, including growth factors (Leof, 2000), cytokines (O'Shea and Murray, 2008), and surface chemistry (Mrksich, 2000). Through intricate signaling networks, individual cells are capable of responding to a wide range of extracellular signals (Osborn and Olefsky, 2012), allowing them to regulate and execute numerous functions in a coordinated manner (Serafini et al., 2015). Cells have evolved sophisticated signaling mechanisms to effectively interpret and translate stimulus-specific information into phenotypic responses, leading to changes in gene and protein expressions (Purvis and Lahav, 2013). These signaling networks often convey diverse signal inputs arising from ligand-receptor interactions, resulting in heterogeneous outputs (Spiller et al., 2010).

**FIGURE 1:**
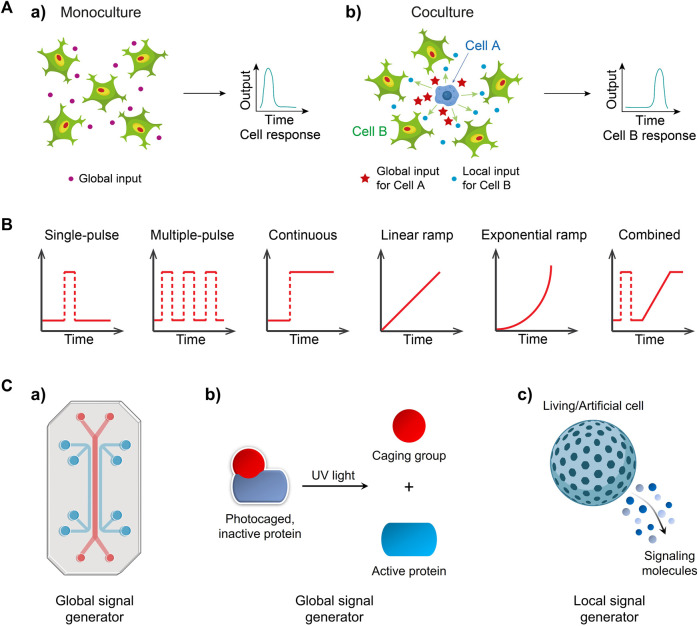
Exploring cellular signaling dynamics in response to global and local inputs can involve the use of various signal generators **(A)** a) Environmental inputs like cytokines are sensed and processed by a group of cells, and then are transformed into an output (e.g., signaling factor activity) b) One cell type (cell A, e.g., macrophage) discerned a global input like bacterial stimuli, and then generated a local input that can be detected by the cocultured neighbors (cell B, e.g., fibroblast) and is thus transformed into a final output Adapted from (Yang et al., 2022) Copyright 2022 CC BY 4.0. **(B)** Temporal input modes observed in biological systems. Input mode/type refers to how signal molecules are applied to cells. **(C)** Engineering global signal generators, including a) a microfluidic system and b) photoactivatable signaling molecules and local generators such as c) a living/artificial cell.

The majority of studies in the field of cell signaling can be broadly categorized into two scenarios: 1) homocellular signaling, which involves signal transduction within identical cell types (monoculture), and 2) heterocellular signaling, which describes signal transmission between two distinct cell types (coculture). In the case of homocellular signaling (Figure 1Aa), a population of identical cells receives an external input from the environment. This global input signal is then processed and interpreted by activating genetically encoded signaling pathways, such as extracellular signal-regulated kinase (ERK) (Lavoie et al., 2020), nuclear factor-kappa B (NF-κB) (Dorrington and Fraser, 2019) and signal transducer and activator of transcription (STAT) (Villarino et al., 2017), leading to an appropriate response (output) induced by the responding cells. In the context of heterocellular signaling (Figure 1Ab), one subpopulation (cell type A) initiates the first response by converting the original environmental input into a signaling mediator. This mediator is then secreted and released to the extracellular space, serving as a local input. The neighboring heterotypic cells (cell type B) receive and transmit the local input signal through internal signaling pathways, ultimately producing a final output. Understanding these signal flows is crucial for unraveling essential biological processes such as cell growth and proliferation (Zhu and Thompson, 2019), immune responses (Brubaker et al., 2015), tumor progression (Yuan et al., 2016), and wound healing (Dekoninck and Blanpain, 2019).

Furthermore, the microenvironment within the body is subject to rapidly changes influenced by various signaling processes. The presence of transient gradients of signaling molecules facilitates cellular communication and regulates cellular functions. For instance, gonadotropin-releasing hormone (GnRH) is secreted in short pulses, activating the synthesis and release of pituitary gonadotropin hormones, thereby regulating reproductive functions (Moenter et al., 1992). Pulsatile flow of ERK signaling at different frequencies plays a crucial role in regulating fundamental cellular processes, including proliferation, differentiation, and cell cycle progression (Sun et al., 2015). A pulsed, strong lipopolysaccharide (LPS) signal triggers rapid and uniform nuclear factor-κB (NF-κB) responses in fibroblasts, while a weak, sustained signal results in varied responses (Kellogg et al., 2015). The dynamic patterns of signaling molecules encompass pulse, continuous, ramp and combined input signals (Figure 1B).

It is widely recognized that dynamic signal processing are ubiquitous in cellular systems. However, understanding how cells interpret these input signals can be challenging. This challenge arises because population-level measurements often mask the heterogeneous behavior exhibited by individual cells, and conventional methods often lack the ability to generate various types of targeted perturbations other than continuous inputs in signaling pathways, for example, signal molecules simply added in well plates continuously stimulate cells. Moreover, observing cellular events in multiple contexts is essential for multi-dimensional understanding of signaling process, as evident in the distinct cellular responses to global and local inputs (Yang et al., 2022). Consequently, there is a growing demand for the development of specific signal generators that enable precise control over defined input modes, thereby allowing investigations into temporal and spatial dynamics of cellular signaling. In this review, we will highlight various signal generators that have been realized using microfluidic systems (Figure 1Ca), photoactivatable signaling molecules (Figure 1Cb), and living/artificial cells (Figure 1Cc) for global or local input control. Furthermore, we will discuss the advantages and limitations of these signal generators and provide insights for their future development.

## Global signal generators for temporal cellular signaling dynamics

Global signal generators provide uniform inputs that allow for the study of both population-averaged and single-cell responses. Currently, the primary methods employed for generating global inputs include microfluidic molecule delivery and the photodeprotection of caged input molecules. Microfluidic systems can provide a wide range of input modes, such as pulse (Blazek et al., 2015; Ryu et al., 2015), continuous (Dettinger et al., 2018; Mudla et al., 2020), sinusoidal (Piehler et al., 2017) and ramping (Song et al., 2018; Mokashi et al., 2019). On the other hand, the range of input types is relatively limited when utilizing photochemical methods (Ryu et al., 2014; Mogaki et al., 2019). In this section, we will discuss the principles of these two methods and explore their applications.

### Global input generation with microfluidics

In the past decade, microfluidics has made remarkable advancements in exploring temporal cellular behaviors (Irimia, 2010; Gao et al., 2012; Kim et al., 2014; Sinha et al., 2018). Microfluidic devices can replicate *in vivo* biological environments with great accuracy and enable high-content analysis of cells. Microfluidics technology offers precise automation and control of analytical functions, enabling high-resolution manipulation of cells and their microenvironments. With these properties, we can modulate cellular signaling pathways to gain insights into mechanisms underlying cell activation, migration, and intercellular communication. Recent studies investigating temporal signaling dynamics using microfluidics-based global input generators are summarized in Table 1.

**TABLE 1:** Summary of microfluidics-based global signal generators for cellular signaling studies.

Cell type	Input molecule	Input type	Application	Reference
NIH3T3 fibroblast	TNFα	Pulse, continuous	Nuclear NF-κB dynamics	Tay *et al.* (2010)
NIH3T3 fibroblast, mouse embryonic fibroblast	platelet-derived growth factor (PDGF)	Pulse	Phosphorylation kinetics of Akt, GSK-3β, p70S6K, S6, Erk1/2, and mTOR	Blazek *et al.* (2013)
NIH3T3 fibroblast	PDGF, insulin-like growth factor (IGF-1)	Pulse	Phosphorylation kinetics of PDGF and IGF-1 receptors, Akt and GSK-3β	Blazek *et al.* (2015)
NIH3T3 fibroblast	Lipopolysaccharide (LPS)	Pulse, continuous	Nuclear NF-κB dynamics	Kellogg *et al.* (2015)
PC 12 cell	Epidermal (EGF), nerve (NGF) growth factor	Pulse, continuous	Nuclear ERK dynamics	Ryu *et al.* (2015)
RAW 264.7 macrophage	LPS	Pulse, continuous	Nuclear NF-κB dynamics, TNF-α secretion dynamics	Junkin *et al.* (2016)
NIH3T3 fibroblast	TNFα	Sine-wave, linear ramping	Nuclear NF-κB dynamics	Piehler *et al.* (2017)
HEK293 B^5^, NIH3T3 cell	EGF	Pulse, linear stepwise ramping	Nuclear ERK dynamics	Song *et al.* (2018)
Murine hematopoietic stem and progenitor cells	Macrophage colony-stimulating factor (M-CSF)	Continuous	Lysozyme M (LysM) gene induction	Dettinger *et al.* (2018)
PC 12 cell	EGF, NGF, fibroblast GF (FGF2)	Pulse, continuous	Nuclear ERK dynamics	Blum *et al.* (2019)
HeLa cells	TNFα	Continuous, linear ramping	Nuclear NF-κB dynamics	Mokashi *et al.* (2019)
HeLa cells	IFNα	Pulse, continuous	IRF9 dynamics, nuclear STAT1 dynamics	Mudla *et al.* (2020)
NIH3T3 fibroblast	TNF, interleukin 1β (IL-1β)	Step, linear/exponential stepwise ramping	Nuclear NF-κB dynamics	Son *et al.* (2021)
K562 cell, NIH3T3 fibroblast	Dimethyl sulfoxide (DMSO), IFNγ	Pulse, continuous	Caspase 3 dynamics, nuclear STAT1 dynamics	Sinha *et al.* (2022)
NIH3T3 fibroblast	IFNγ	Pulse, continuous	Nuclear STAT1/2 dynamics	Yang *et al.* (2022)

**FIGURE 2:**
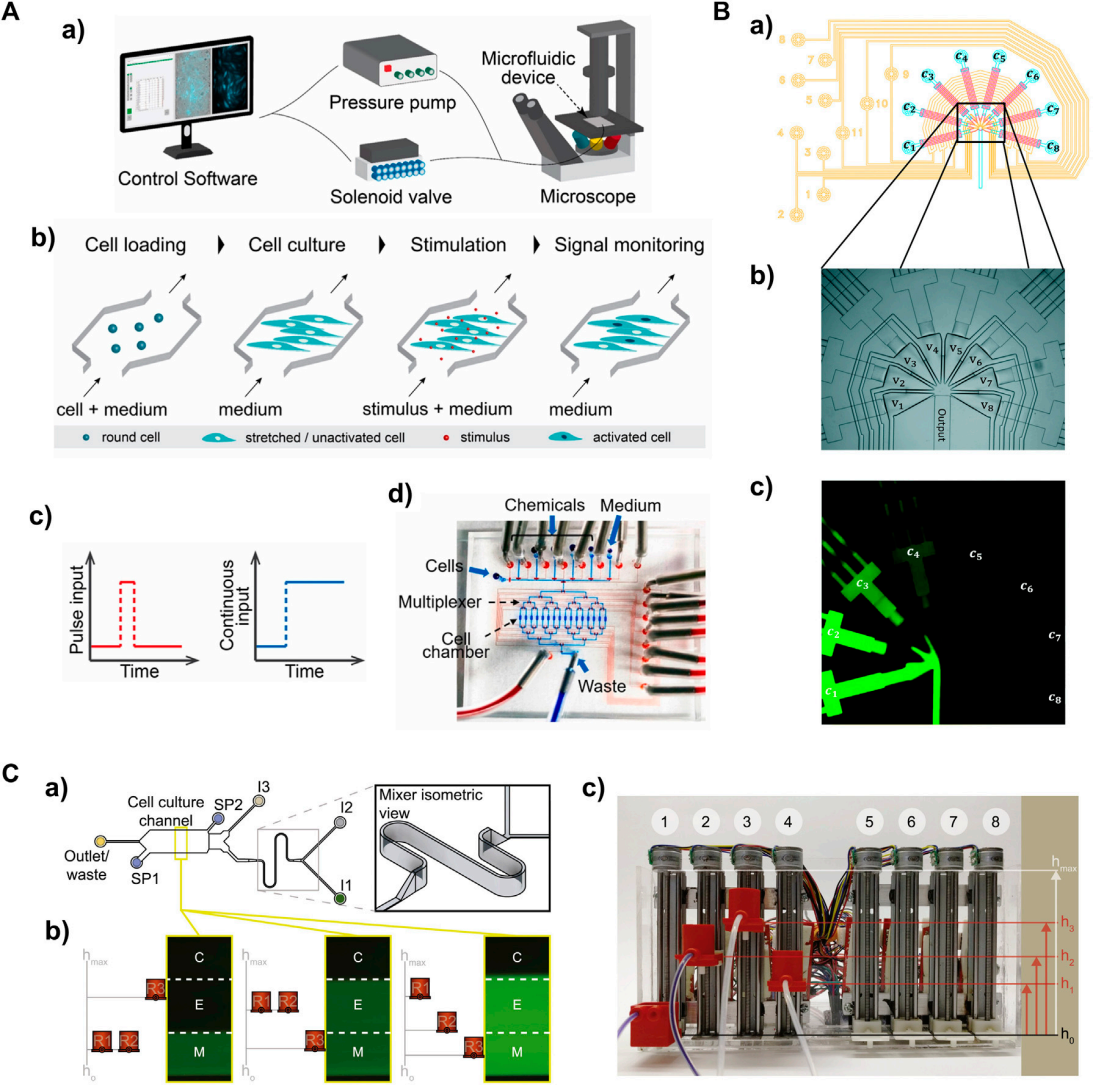
Microfluidic-based global signal generators for studying temporal cellular signaling dynamics **(A)** A microfluidic system capable of producing a pulse or continuous input to cells. a) The entire system. The delivery of defined input types are controlled by a pressure pump and solenoid valves. Outputs are acquired via time-lapse live-cell microscopy. b) The workflow to measure transcription factor activity in adherent cells expressing fluorescence reporters. c) One-pulse and continuous input profiles. d) The multilayer microfluidic device with control layer in red (e.g., microvalves) and flow layer in blue (e.g., cell chambers). Adapted from (Yang et al., 2022) Copyright 2022 CC BY 4.0. **(B)** A PDMS signal generator capable of producing sinusoidal inputs. a) Design of the signal generator module; control layer in yellow, flow layer in blue and red. b) The injector junction with triangular converging valves v1v8 in the multilayer microfluidic device. c) The operating signal generator involves mixing fluorescent dye solutions of different concentration (c1c8). Reproduced with permission from Piehler et al. (2017). Copyright 2017 The Royal Society of Chemistry. **(C)** A gravity pump-integrated microfluidic system that enables analog control of input strength. a) Top view of the dynamic stimulation device. b) Flow rates through the inlets (I1, I2 and I3) are controlled by hydrostatic pressure differences between corresponding reservoirs (R1, R2, and R3) and the outlet. c) The “gravity pump” comprises eight vertically mounted stepper motors with screw-nut platforms and an Arduino microcontroller to control platform heights. Adapted from (Mokashi et al., 2019) Copyright 2019 CC BY 4.0.

A typical microfluidic platform for studying cellular signaling consists of a microfluidic device, a custom software control system, a pressure pump, solenoid valves, and a live-cell imaging microscope (Figure 2Aa) (Yang et al., 2022). The low cost and biocompatibility of polydimethylsiloxane (PDMS) make it ideal for rapid fabrication of microfluidic devices using soft lithography, which has led to the development of various microfluidic designs (Sia and Whitesides, 2003). PDMS is permeable to gases, allowing replication of artificial cellular microenvironments *in vitro*, and its flexibility enables easy integration of membrane valves and pumps to create intricate networks of microchannels (Thorsen et al., 2002). This enables full automation of protocols using programming software (White and Streets, 2018; Kehl et al., 2021). The PDMS microfluidic device consists of a bottom flow layer for sample loading and a top control layer for valve actuation (Figure 2Ad). The membrane valves can be pneumatically/hydraulically actuated using a pressure pump and solenoid valves (Brower et al., 2018; Watson and Senyo, 2019). This precise control allows for cell seeding, medium exchange and input delivery for studying cellular signaling (Figure 2Ab).

Various input profiles can be defined and implemented with high precision (Table 1). Different input types can be achieved by controlling input amplitude and duration through opening and closing the embedded membrane valves (the layer in red in Figure 2Ad). The typical input modes of cytokine interferon γ (IFNγ), such as pulse and continuous (Figure 2Ac), were applied to perturb the activity of transcription factor STAT1 in single fibroblasts or populations (Yang et al., 2022). Distinct STAT1 activation dynamics were observed between one-pulse and continuous IFNγ treatment. This indicates that STAT1 activation can be temporally modulated by introducing different temporal stimulation profiles. Another transcription factor, NF-κB, displayed activation and oscillation dynamics when subjected to a continuous cytokine input of tumor necrosis factor *a* (TNFα). Applying a stepwise ramping input of TNFα or interleukine-1β (IL-1β) to fibroblasts revealed that the activity of the NF-κB signaling pathway correlated with the rate of change in cytokine concentrations rather than the absolute cytokine concentrations. In addition, the implementation of sinusoidal inputs was realized using a multiple-layer PDMS device with eight triangular converging valves (Figure 2B). Fibroblasts stimulated with sinusoidal TNF inputs showed characteristic NF-κB nucleocytoplasmic oscillations with great heterogeneity in single-cell responses (Piehler et al., 2017). While the duration and amplitude of inputs can be readily controlled in membrane valve-embedded PDMS microfluidic devices, implementing ramping analog inputs (Song et al., 2018; Son et al., 2021) in the PDMS devices presents a challenge. Recently, a gravity-driven flow has been achieved in a microfluidic device with high-aspect-ratio channels controlled by a gravity pump (Figure 2C) (Mokashi et al., 2019). This fully analog system is capable of producing arbitrarily complex patterns of input signals. Ramping input of TNFα led to increased NF-κB dynamics in a fraction of cells compared to those showing qualitatively different NF-kB responses to continuous stimulation. These observations demonstrate the ability of microfluidic systems to create various defined input types that can induce distinct cellular responses, which is crucial for discovering underlying mechanisms of temporal cellular signaling.

Microfluidic devices integrated with cell traps have significantly advanced research by providing opportunities to study single cells and gain insights into their signaling dynamics. These devices allow the isolation of individual cells, which is often challenging with other technologies. The designs for single-cell analysis typically utilize unique geometric structures, such as pillar-like (Junkin et al., 2016; Sinha et al., 2022) and V-type (Rho et al., 2016) valves. A microfluidic device with pillar-like traps was developed for quantitative analysis of single-cell immune dynamics (Junkin et al., 2016). With these traps, single macrophages were isolated and exposed to different input types, including a single-pulse, continuous, and repeated pulses of lipopolysaccharide (LPS), separately. The dynamics of TNFα secretion in single macrophages was found highly heterogeneous and surprisingly uncorrelated with the dynamics of NF-κB, the transcription factor that controls TNFα production. Additionally, simulation analysis revealed that a trap with an optimal geometric structure can achieve single-cell trapping with high precision (Sinha et al., 2022). These global signal generators have facilitated the exploration of temporal signaling dynamics (such as transcription factor dynamics) in single cells encountering defined environmental perturbations, enriching our understanding of how extracellular signal inputs were interpreted by single cells, and how these dynamics affect their downstream signaling events, such as cytokine secretion.

### Global input generation with photoactivation

While microfluidic systems can be used to deliver global input signals to cells, they may result in a delay of seconds for inputs among different locations within cell culture, thereby posing a challenge for investigating fast signaling events. The activity of global input molecules can be suppressed and controllably activated by stimuli, such as light irradiation (Klan et al., 2013). This occurs because every signal is transmitted into cells upon binding of input molecules to specific receptors. Recently, several photoactivation-based methods have been reported (Ryu et al., 2014; Mogaki et al., 2019; Perdue et al., 2020), which allows for spatial control of signal molecules using light. Due to the simplicity and ease of light irradiation, efforts have been made to develop different strategies for small molecules and proteins.

**FIGURE 3:**
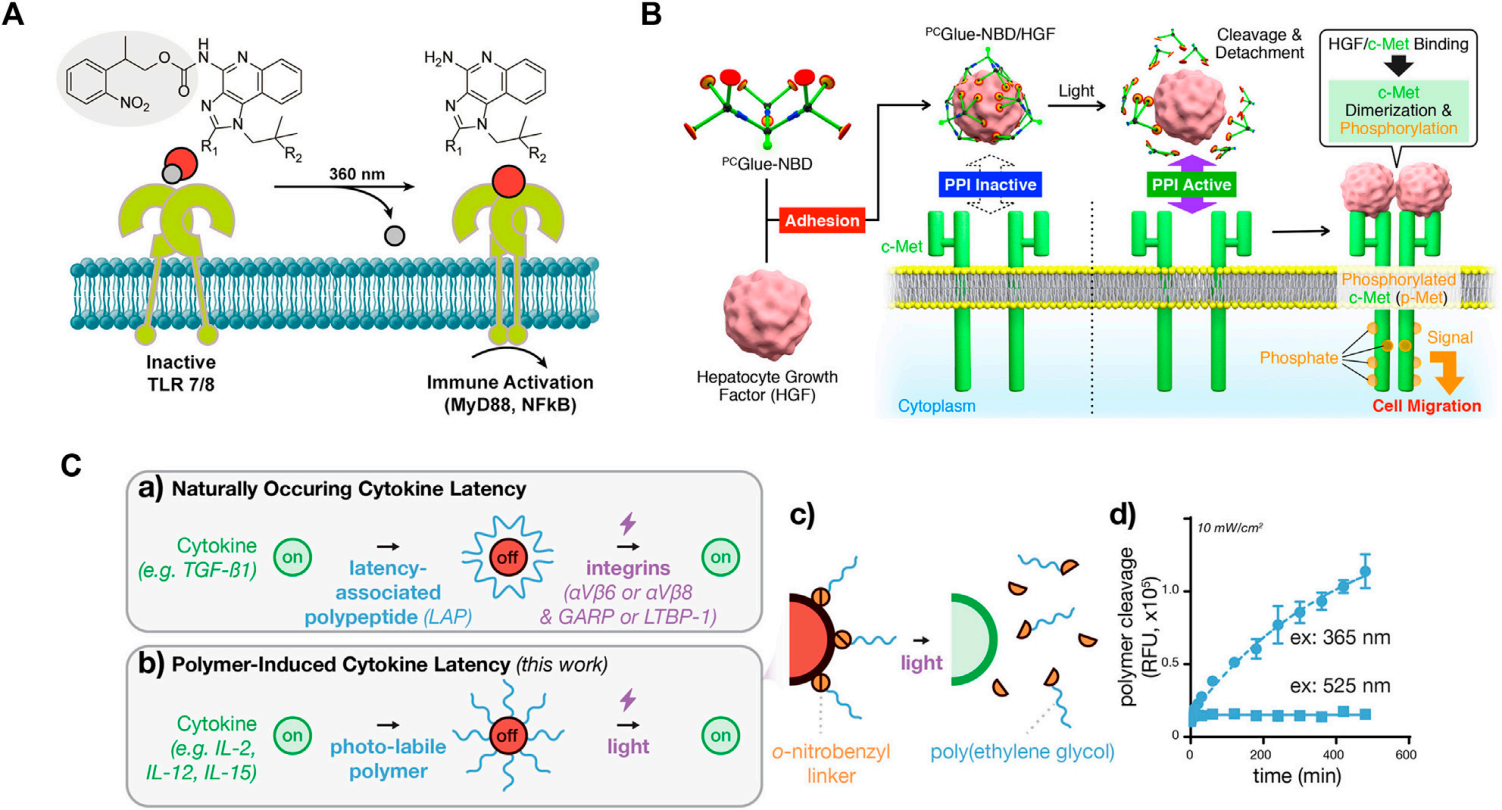
Photoactivation of signaling molecules as a global input for cellular signaling study **(A)** Photodeprotection of caged small-molecule agonist for controlled TLR7/8 activation. Reproduced with permission from (Ryu et al., 2014) Copyright 2014 American Chemical Society. **(B)** Photodeprotection of dendritic molecular glue-caged hepatocyte growth factor (HGF) induces cell migration. Reproduced with permission from (Mogaki et al., 2019) Copyright 2019 American Chemical Society. **(C)** Photodeprotection of polymer-caged cytokine for controlled T cell proliferation. The 20 kDa poly(ethylene glycol) polymer chains appended to cytokine lysine residues via o-nitrobenzyl groups, which are rapidly cleaved by blue LED light, as measured by cleavage-induced fluorescence dequenching. Reproduced with permission from (Perdue et al., 2020) Copyright 2020 American Chemical Society.

For small molecule inputs, they can be caged by a photocleavable group, such as the 2-nitrobenzyl group, to inhibit their activity. Two small molecules, Imiquimod (R837) and Resiquimod (R848), which are agonists of Toll-like receptor 7 (TLR7) and TLR7/8, respectively, were conjugated with the photo-protecting group carbamate of 2-(2-nitrophenyl)-propyloxycarbonyl (NPPOC) to suppress their spatial activity. Irradiation with 360 nm UV light deprotected these small-molecule agonists, triggering signal transmission and NF-κB pathway activation in cells (Figure 3A) (Ryu et al., 2014). Similarly, a TLR4 agonist, pyrimido [5,4-b]indole, was photocaged at a position critical for receptor binding by protecting the indole nitrogen with 6-nitroveratryloxycarbonyl (NVOC). Upon exposure to UV light, the agonist was uncaged and activated NF-κB (Stutts and Esser-Kahn, 2015). In addition to photocaging methods, photoresponsive conformational switches of small molecule inputs can also reversibly change their activity. A photoswitchable Pam_3_CS_4_ derivative–P10 was synthesized to control the activation of the TLR1/2 signaling pathway. The ground-state *trans*-P10 can activate antigen-presenting cells (APCs) by promoting TLR1/2 heterodimerization. In the presence of UV irradiation, *trans*-P10 is converted to *cis*-P10, which reduces the activities of APCs by impeding the TLR1/2 heterodimerization (Hu et al., 2020). These methods offer the potential to regulate immune activation and inflammation.

For macromolecule inputs, such as growth factors and cytokines, it is challenging to directly modify them with photo-protecting groups. A dendritic molecular glue, ^PC^Glue-NBD, carries multiple guanidinium ion (Gu^+^) pendants. This functional molecular glue can strongly adhere to the target protein, hepatocyte growth factor (HGF), and cover the region for protein-protein interactions (PPIs) on their surfaces. The PPIs are inactive, suppressing cellular signaling. Upon irradiation with UV light, ^PC^Glue-NBD is photocleaved, reducing the multivalency for the adhesion. Consequently, uncaged HGFs regains its intrinsic PPI affinity toward c-Met, leading to pathway activation and cell migration (Figure 3B) (Mogaki et al., 2019). This study demonstrates a universal strategy for suppressing the activity of macromolecule inputs, holding great promise for controlling protein input-mediated signaling.

Another strategy that can reversibly suppress protein activity is chemical modification with photolabile polymers (Perdue et al., 2020). Cytokines such as human interleukin-2 (IL-2), IL-15, and mouse scIL-12 were caged with polyethylene glycol (PEG) -conjugated with a 2-nitrobenzyl linker. UV irradiation photocleaved the 2-nitrobenzyl linkers, causing PEG to detach and thus restoring the activity of cytokines (Figure 3C). The magnitude and the duration of cytokine signaling can be tuned on demand, with high spatial resolution. This approach is also applicable to a range of additional cytokine or chemokine proteins. Although the activity of proteins is initially suppressed, cells still initiate a delayed response to the photocaged cytokine proteins. In contrast, the uncaged cytokine proteins induce a significantly faster response. These findings demonstrate the controllability of cytokine signaling latency using light. Although this strategy does not directly control the activation, continuous efforts may lead to improvements for this purpose.

Several recent light-controlled global signal generators are summarized in Table 2. The effectiveness of photoactivation methods relies on the photoresponsive groups or linkers used. Current methods are based on the use of short-wavelength light, such as UV irradiation. The should be noted that pathways sensitive to UV light may be activated or interfered with (Devary et al., 1993; Li and Karin, 1998; O'Dea et al., 2008). Due to the strong phototoxicity of UV light, cells may not survive prolonged exposures. Exploring alternative photocleavable groups responsive to long wavelengths of light can be a promising direction for controlling cellular signaling (Wegener et al., 2017).

**TABLE 2:** Summary of light-controlled global signal generators for cellular signaling studies.

Cell type	Input molecule	Photosensitive moiety	Application	Reference
Bone marrow-derived dendritic cell (BMDC)	TLR7 agonist, Imiquimod (R837), TLR7/8 agonist, Resiquimod (R848)	Carbamate of 2-(2-nitrophenyl)-propyloxycarbonyl (NPPOC)	NF-κB activation, CD40 expression, IL-12, TNF-α and IL-6 secretion	Ryu *et al.* (2014)
NIH3T3 fibroblast	TLR4 agonist, pyrimido [5,4-b]indole	6-nitroveratryloxycarbonyl (NVOC)	NF-κB activation	Stutts and Esser-Kahn (2015)
Namalwa cell	TLR9 agonist, CpG oligonculeotide	Nitropiperonyloxymethyl (NPOM)	IL-6 expression	Govan *et al.* (2015)
BMDC	TLR2/6 agonist, Pam_2_CSK_4_	NPPOC	*In vivo* NF-κB activation, upregulation of nfkb1, cd34, cd28 and ccr7 expression	Ryu *et al.* (2017)
T lymphocyte	Moth cytochrome c_88-103_ (MCC), ovalbumin_257-264_ (OVA)	Nitrophenylethyl (NPE)	Diacylglycerol (DAG) accumulation, centrosome reorientation, and Grb2 microcluster formation	Sanchez and Huse (2018)
THP-1 cell and RAW 264.7 macrophage	Pam_3_CSK_4_ derivative–P10	The metastable *cis*-P10 is converted to its thermally stable *trans* configuration	NF-κB activation, upregulation of CD80, CD86, CD40 expression, and IL-1β, TNF-α, IL-6, IL-12 secretion	Hu *et al.* (2020)
Human prostate carcinoma DU145 cell	Hepatocyte growth factor (HGF)	Molecular glue ^PC^Glue-NBD, carrying nine Gu^+^ pendants and butyrate-substituted NVOC (^BA^NVOC) linkages	Cell migration	Mogaki *et al.* (2019)
CTLL-2 T cell	Human IL-2, mouse scIL-12	Polyethylene glycol (PEG) modified with 2-nitrobenzyl linker derivatives	T cell proliferation, OVA_257−264_ antigen-specific T cell activation, and STAT5 activation	Perdue *et al.* (2020)

## Local signal generators for spatiotemporal cellular signaling dynamics

While global signal generators can be used to explore temporal signaling dynamics, probing spatial cellular behaviors remains a challenge. *In vivo*, signal sender cells are located within cell populations and transmit signals in either a two-dimensional (2D) or one-dimensional (1D) path (Frank and Tay, 2015). The construction of such signaling patterns requires precise spatial and temporal control over the stimulation of sender cells, referred to as local signal sources, without interfering with receiver cells. Recent methods have focused on leveraging living or artificial cells as local signal generators.

### Local input generation with living cell senders

Living cell senders serve as natural local signal sources due to their ability to secrete signals within the physiological range. A critical step is the activation of sender cells. This section will discuss recent strategies for the controlled activation of living sender cells, including pre-stimulation, microfluidics-assisted stimulation, photocaged global input, and optogenetic activation. Recent studies on living cell-based local input generators are summarized in Table 3.

**TABLE 3:** Summary of living cell sender-based local signal generators for cellular signaling studies.

Sender/receiver cell type	Method of local input activation	Local input molecule	Application	Reference
T cell/T cell	Pre-stimulation of sender cells with phorbol myristate acetate (PMA) and ionomycin	IL-2	STAT5, FoxP3 activation in receiver cells	Oyler-Yaniv *et al.* (2017)
RAW 264.7 macrophage/NIH3T3 fibroblast	Microfluidic delivery of LPS to stimulate sender cell	TNF	Nuclear NF-κB dynamics in both sender and receiver cells	Frank and Tay (2015), Son *et al.* (2022)
RAW 264.7 macrophage/HEK293 cell	Microfluidic delivery of LPS to stimulate sender cell	TNFα	Nuclear NF-κB activation in receiver cells	Watson *et al.* (2022)
HES3 cell/HES3 cell	Microfluidic delivery of bone morphogenetic protein 4 (BMP4) to a colony	BMP4	MIXL1, T, SOX17, CDX2 expression in receiver cells	Manfrin *et al.* (2019)
Tumor cell/stromal cell	Input molecules secreted in the normal culture in Matrigel-fulfilled microfluidic device	TGF-β1	α-smooth muscle actin (α-SMA) expression in receiver cells	Fang *et al.* (2021)
BMDC/RAW Macrophage, HEK293 cell, fibroblast	Light-activated NPPOC-modified TLR2/6 agonist	TNF	NF-κB activation, TNF secretion in receiver cells	Mancini *et al.* (2015)
Opto-SOS/WT NIH3T3 fibroblast	Light irradiation on sender cells	IL-6	ERK activation in sender cells, STAT3 activation in receiver cells	Toettcher *et al.* (2013)
NIH3T3 fibroblast/NIH3T3 fibroblast	4-hydroxytamoxifen (4-OHT)-induced production of Sonic hedgehog (SHH) in sender cells	SHH	Reconstitution of SHH signaling gradients for quantitative analysis of spatiotemporal patterning dynamics in receiver cells	Li *et al.* (2018)

To construct a local signaling model, sender cells can be pre-stimulated before cocultured with receiver cells. The activated sender cells become local signal sources, secreting input signals in limited areas. Recently, a diffusion-consumption model has been created using the pre-stimulated T cells as the living senders to produce IL-2, which stimulates surrounding T cell receivers (Oyler-Yaniv et al., 2017). Immunofluorescence staining revealed the generation of microdomains of STAT5-activated T cells around local IL-2 sources. Although this method is operationally simple and can be easily applied to investigate the activation status of cells within local regions, it is challenging to measure the signaling dynamics of receiver cells. The challenge lies in precisely controlling the secretion of cytokine from living sender cells, which makes it difficult to track the origin of signal propagation and transduction in receiver cells.

**FIGURE 4:**
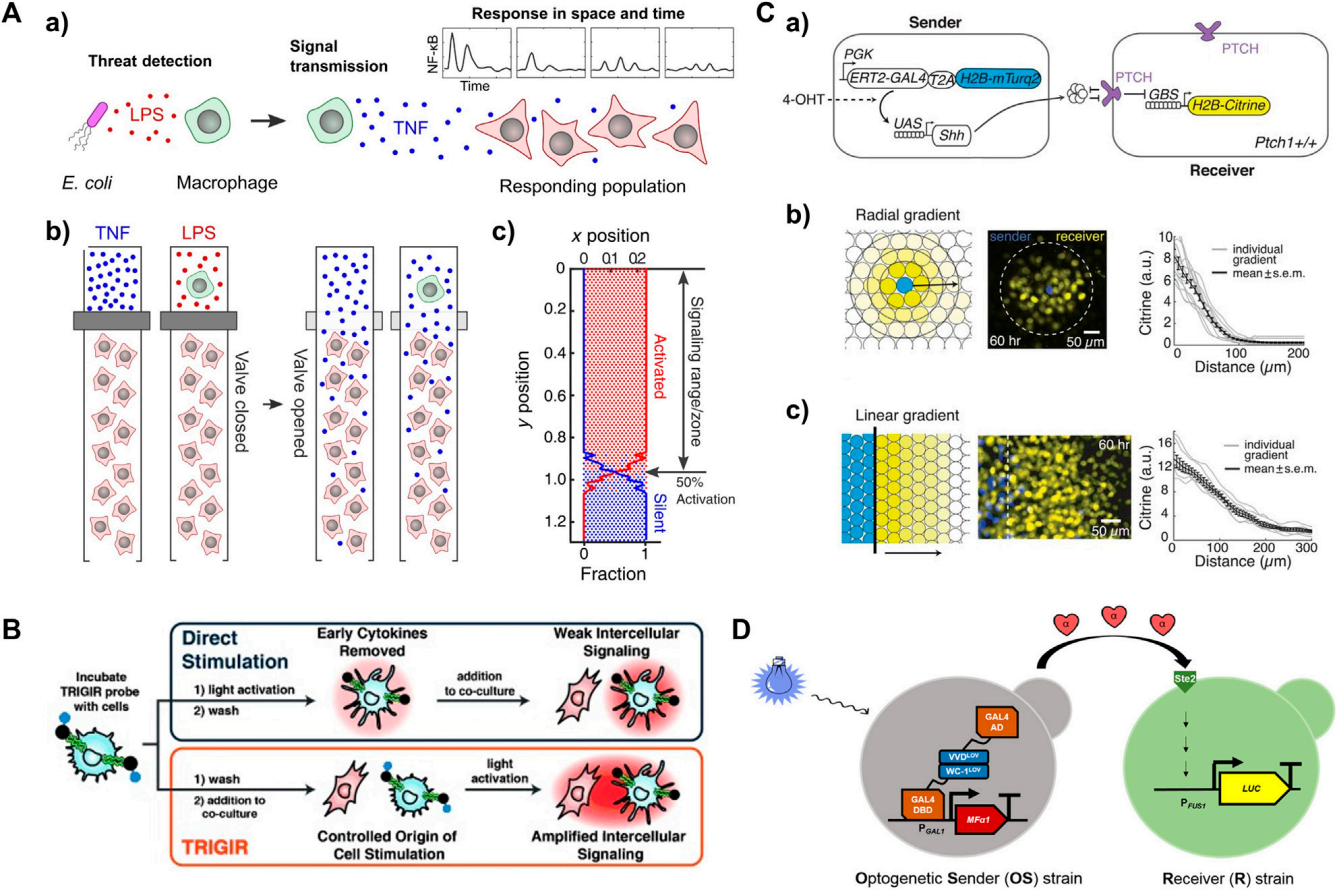
Activated living cells as a local input source for studying spatiotemporal signaling dynamics in receiver cells **(A)** a) Lipopolysaccharide (LPS)-infected macrophages induce immune responses locally. b) The use of valve to control the propagation of a local signal (e.g., cytokine or an active macrophage) to neighboring fibroblasts in microfluidic device. c) Fibroblast activation at each location derived from simulation. Reproduced with permission from (Son et al., 2022) Copyright 2022 CC BY-NC 4.0. **(B)** Light-activated dendritic cell serve as a local signal generator to propagate inflammatory information to neighboring immune cells. Reproduced with permission from (Mancini et al., 2015) Copyright 2015 WILEY-VCH Verlag GmbH & Co. KGaA. **(C)** a) Sender and receiver cell lines for reconstituting morphogen Sonic hedgehog (SHH) signaling gradient upon induction with 4-hydroxytamoxifen (4-OHT). Reconstituting SHH signaling gradients in b) radial and c) linear geometries. Reproduced with permission from (Li et al., 2018). Copyright 2018 The American Association for the Advancement of Science. **(D)** Optogenetic control of the secretion of α-factor pheromone by Optogenetic Sender (OS) strains, which serves as a local input that triggers the mating pathway, leading to the expression of a luciferase reporter gene under the control of the FUS1 pheromone-responsive promoter in Receiver (R) strains. Reproduced with permission from (Rojas and Larrondo, 2022). Copyright 2022 American Chemical Society.

Valve-integrated microfluidic devices enable the coculture of a single sender cell (e.g., macrophage) and a population of receiver cells (e.g., fibroblasts), as well as the control of signal propagation (Frank and Tay, 2015; Son et al., 2022; Watson et al., 2022). These devices consist of connected channels with a separation valve between macrophages and fibroblasts, (Figure 4Ab), creating a 1D signaling axis (Frank and Tay, 2015; Son et al., 2022). Dynamic LPS inputs can be delivered to single macrophages, initiating NF-κB pathway activation and TNFα secretion, which becomes a local TNFα source. By opening the separation valve, TNFα transmits along the channels in a wave-like propagation, initiating temporal and spatial responses of NF-κB in the cocultured fibroblasts (Figures 4Aa–c). This model enables control of local signal flow by opening and closing the separation valve. A microfluidic device facilitating unidirectional intercellular communication can avoid crosstalk and interference between sender and receiver cells (Fang et al., 2021). The device consists of two separated half-ellipse-shaped chambers for different cell cultures, which are mixed in Matrigel and loaded into the left and right chambers, respectively. Matrigel and physical barriers restrict the medium flow to form a unidirectional signal flow from sender to receiver cells. Additionally, the device allows the analysis of functional signals secreted by sender cells via a signal-blocking inlet.

Although 1D signaling models have been realized for spatiotemporal signaling studies, the output information is still limited because *in vivo* local signaling patterns are typically 2D or 3D. A 2D model of developmental signaling center has been created in microfluidic device (Manfrin et al., 2019). Localized morphogen signaling sources were generated upon treatment with an input signal of bone morphogenetic protein 4 (BMP4), resulting in the formation of morphogen gradients along human pluripotent stem cell (hPSC) colonies. The hPSCs exhibited spatially differential expression of MIXL1, T, SOX17 and CDX2 genes, demonstrating spatiotemporally controlled morphogen signaling gradients. This study shows the possibility of constructing a 2D signaling model in a microfluidic device, provided that a global signal does not stimulate receiver cells.

Photocaging and photoactivation strategies can also be used to control the activation of sender cells in a 2D signaling model. For example, a light-controlled immunostimulant probe that can photosensitize immune cells was synthesized to control the origin of inflammation (Mancini et al., 2015). This probe, a photocaged TLR agonist modified with a 2-nitrobenzyl linker, can tag and remotely induce a guided immune response (TRIGIR) (Figure 4B). With light irradiation, the TRIGIR probe is uncaged after the photocleavage of the 2-nitrobenzyl linker, functioning as a photoactive immunopotentiator to activate TLR signaling and inflammation pathways. In a coculture environment, the photoactivated TRIGIR probe controllably activates bone marrow-derived dendritic cells (BMDCs) as a global input. The cocultured fibroblasts are further activated upon receiving local input signals from the activated BMDCs, thereby initiating TNF secretion.

Synthetic biology offers powerful tools such as chemogenetic (Keifer et al., 2020; Tsai et al., 2021; Raper and Galvan, 2022) and optogenetic (Tischer and Weiner, 2014; Zhang and Cu, 2015; Leopold et al., 2018; Hongdusit et al., 2020; Farahani et al., 2021; Kramer et al., 2021) techniques to control the activity of signaling proteins in living cells. By leveraging these tools, it is possible to control the activation of living sender cells that have been transfected with chemogenetic or optogenetic response elements. An example involved the production of morphogen Sonic Hedgehog (SHH) by a sender cell line under the control of the chemical 4-hydroxytamoxifen (4-OHT) (Figure 4Ca) (Li et al., 2018). The SHH signaling gradients resulted in radial and linear activation geometries in neighboring receiver cells, as evidenced by the expression of nuclear-localized Histone 2B (H2B)-Citrine fluorescent protein (Figure 4Cb). This finding showcases the ability to achieve localized signal sources through the utilization of synthetic circuits controlled by chemical inputs. The optogenetic approach allows the construction of an intercellular communication model in which local signal generation can be precisely controlled through light irradiation on living sender cells. The design of an opto-SOS system enabled the controlled initiation of signaling protein Ras activation, nuclear translocation of Erk2, and secretion of IL-6 family cytokines (Toettcher et al., 2013). The observation of STAT3 nuclear translocation in receiver cells confirmed the propagation of IL-6 from the signaling senders. Notably, 2 hours of light irradiation on sender cells led to STAT3 activation in the receivers, whereas two separate 1-h light irradiations with an interval did not produce the same effect. In another study, an optogenetic intercellular system was implemented in the budding yeast Saccharomyces cerevisiae. This system involved controlling the production of α-factor pheromone through blue light irradiation of Optogenetic Sender (OS) strains, subsequently leading to luciferase induction in the Receiver (R) strains (Figure 4D) (Rojas and Larrondo, 2022). These studies demonstrate the versatility of optogenetic tools in the introducing different types of local inputs that lead to distinct fate decisions in receiver cells.

### Local input generation with artificial cell senders

**FIGURE 5:**
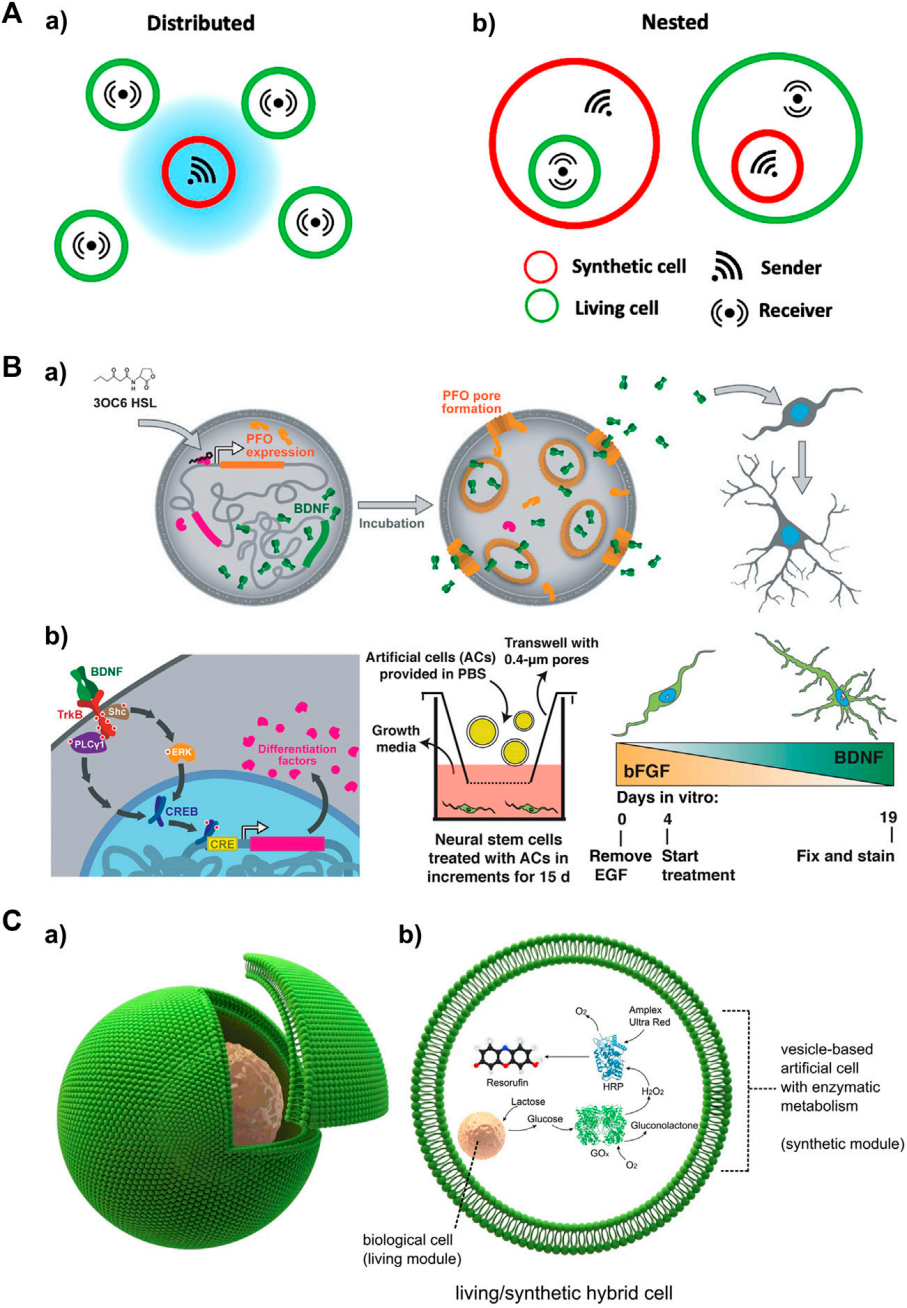
Activated artificial cells as a local input source for studying spatiotemporal signaling dynamics in receiver cells **(A)** Schematic depicting different types of localized signaling in artificial/living cell consortia, including local signaling among a) distributed and b) nested cell populations. Adapted from (Mukwaya et al., 2021) Copyright 2021 CC BY 4.0. **(B)** Small molecule-triggered signaling in an artificial cell as a local signal source to drive neural differentiation. a) 3OC6 HSL induced PFO expression and pore formation, along with BDNF release, which subsequently leads to the differentiation and maturation of mNS cells. b) Left: Signaling between artificial cells and mNS cells. Middle: Artificial cells were incubated with mNS cells in a transwell. Right: BDNF secretion gradually increased over the course of artificial cell treatment (days 4–19). Reproduced with permission from (Toparlak et al., 2020). Copyright 2020 CC BY 4.0. **(C)** Artificial/Living hybrid cells. a) a biological cell encapsulated inside a vesicle-based artificial cell. b) The encapsulated cell functions similarly to an organelle within the vesicle reactor. It processes chemical elements, which are subsequently metabolized downstream by a co-encapsulated synthetic enzymatic cascade in the vesicle. Reproduced from (Elani et al., 2018). Copyright 2018 CC BY 4.0.

Artificial cells, also known as synthetic protocells, are designed to replicate the structures and functions of living cells. These cell mimics provide a valuable tool for studying intercellular communications with minimal interference from cellular complexity, such as diverse secretion levels and rates of signal molecules. Additionally, artificial cells offer advantages in controlling the release of local input signals compared to living cells, which opens up new possibilities for various applications. In the context of local signal generation, artificial cells can be engineered to replace living cells as local signal sources. While several studies have investigated communication between artificial cells (Niederholtmeyer et al., 2018; Aufinger and Simmel, 2019; Joesaar et al., 2019; Karoui et al., 2022), the interaction between artificial and living cells has received less attention. This section will introduce three types of signaling models, namely, paracrine, contact-dependent, and embedded signaling, in the context of artificial-living cell communities (Figure 5A).

Paracrine signaling involves the transmission of signals over short distances, eliciting diverse responses in receiver cells. Artificial cells with biocompatibility can be cocultured with living cells to deliver local input signals via paracrine signaling. A recent development induces an artificial cell system that integrated a brain-derived neurotrophic factor (BDNF) and perfringolysin O (PFO) gene expression construct (Toparlak et al., 2020). This system allows for the controlled activation of both genes using *N*-3-oxohexanoyl homoserine lactone (3OC6 HSL). In the presence of 3OC6 HSL, both PFO and BDNF are produced, and BDNF is released through formed PFO pores. In a coculture system, the artificial cells responded to 3OC6 HSL, releasing BDNF that subsequently drives the differentiation of mouse embryonic stem cell-derived neural stem (mNS) cells (Figure 5B). Communication between artificial cells and engineered HEK293T cells has also been established through the addition of 3OC6 HSL. The released BDNF induces GFP expression in the HEK293T cells. These results demonstrate the suitability of artificial cells in delivering paracrine signals as substitutes for biological cells.

Nested (or embedded) architectures involving artificial and living cells provide non-native signaling configurations (Figure 5Ab). In this construct, artificial cells are embedded within living cells, allowing for the exploration of signaling events initiated inside the system, such as antiviral innate immune signaling (Seth et al., 2006). Although this approach has been relatively less explored in current studies, it holds promise for future applications. For example, micron- or submicron-sized artificial cells loaded with viral DNA/RNA can be endocytosed by living cells, mimicking nested communication during viral infection. The viral DNA/RNA can then be released, triggering the retinoic acid-inducible gene I (RIG-I) and melanoma differentiation-associated gene 5 (MDA5) pathways, as well as NF-κB pathway (Brisse and Ly, 2019; Rehwinkel and Gack, 2020; Onomoto et al., 2021). Another possible configuration involves living cells embedded within an artificial cell, enabling a cellular bionics approach where living cells can function as organelle-like modules. A recent study presented a cellular bionic system consisting of a single host lipid vesicle-based artificial cell encapsulating colon carcinoma cells, and established an embedded glucose oxidase (GOx)/horseradish peroxidase (HRP) enzyme cascade (Figure 5C) (Elani et al., 2018). This localized communication was initiated by the production of glucose (Glc) upon stimulation of the cancer cells with lactose pre-loaded in the artificial cell. This innovative approach has the potential to uncover more signaling mechanisms underlying nested communication.

## Advantages and limitations of current global and local signal generators

Global and local signal generators have been utilized in cellular signaling studies to gain insights into the activation of signaling pathways and dynamics of signaling proteins. Each type of generators has its own set of advantages and limitations, which will be discussed in this section (Table 4).

**TABLE 4:** Comparison of different signal generators in terms of their input types, advantages and limitations.

	Potential input types	Advantages	Limitations
Global Signal Generators			
Microfluidics-based	Pulse, continuous, ramping	Able to implement various input modes	Shear stress might affect quantification of fluorescence-labelled signaling proteins
		Input is native molecule with known concentration	Challenging to deliver input to suspension cells
Photoactivation-based	Continuous	Easy to execute light irradiation	Chemical modification of input molecules may lower down the activity of protein input
			Long time exposure to short wavelength of light may be harmful to cells
Local Signal Generators (Living Cell Senders)			
Pre-stimulation-based	Wave	No need of extra global input delivery or modification	Challenging to control the origin of signal propagation
Microfluidics-based	Wave	Input is native molecule with known concentration	Challenging to deliver input to suspension cells
			2D signaling models require the inputs that are insensitive to receiver cells
Photo-deprotection-based	Wave	Local input is controllable	The receiver cells must be insensitive to the stimuli
			Prolonged exposure to short wavelength of light may be harmful to sender cells
Optogenetics-based	Wave, continuous	Local input is controllable	The specificity of the expression patterns of the optogenetic probes relies on the availability of the appropriate promoter/enhancer sequences
		Photosensitive elements responsive to long wavelength of light can be applied to activate sender cells	
Local Signal Generators (Artificial Cell Senders)			
Artificial cell-based	Wave, continuous	Local input is controllable	Challenging to quantify the input molecules released from the artificial cells
		Exposure with short wavelength of light does not affect non-living sender cells	

Microfluidic systems have been widely used as global signal generators for investigating temporal signaling dynamics (Tay et al., 2010; Song et al., 2018; Mokashi et al., 2019; Yang et al., 2022). These systems allow for precise delivery of native molecules, including cytokine proteins, to cell cultures. With the ability to control input amplitude and duration, microfluidic systems can implement various input types, such as pulse, continuous and ramping, to capture dynamic information about cellular behaviors and gain insights into signaling mechanisms. However, microfluidic systems also have limitations. The perfusion of input molecules into cell chambers can generate strong shear stress, which may affect cell morphology. Cells sensitive to shear stress may shrink (Yang et al., 2022), altering the fluorescence intensity of the nucleus, cytoplasm and the entire cell, thus interfering with the quantitation of signaling proteins. To mitigate this influence, optimization of microchannel geometry, size, and pump pressure is necessary. Additionally, flow-based input delivery is primarily suitable for adherent cells, as keeping suspension cells stationary during perfusion in a microfluidic device is challenging.

Another approach of generating global inputs involves light irradiation to induce the photodeprotection of caged input molecules (Ryu et al., 2014; Stutts and Esser-Kahn, 2015; Ryu et al., 2017). This method addresses the limitations of shear stress and the challenge of handling suspension cells in microfluidic devices. Light irradiation allows for cell experiments to be performed in commercialized well plate, eliminating the need for complex microfluidic device fabrication and setup. However, chemical modification of photocaged groups to input molecules relies heavily on organic synthesis, which may inactivate proteins. To overcome this limitation, proteins can be caged with dendritic molecular glue ^PC^Glue-NBD (Mogaki et al., 2019) or PEG conjugated with 2-nitrobenzyl linkers (Perdue et al., 2020) and photodeprotected with UV irradiation. Nevertheless, prolonged UV light exposure can be phototoxic to cells. Additionally, this approach primarily supports continuous inputs, as the input molecules are not removed from cell cultures after light irradiation.

Local signal sources can be established using either living or artificial cell senders. Various strategies have been employed to control the activation of sender cells. A simple 2D signaling model can be constructed by coculturing pre-stimulated living sender cells with receiver cells (Oyler-Yaniv et al., 2017). This method allows for the investigation of interesting pathways without the need for additional delivery or chemical modification of global input molecules. However, controlling the origin of the local signal source is challenging since the local input molecules start propagating during the pre-stimulation process. Thus, this method is more suitable for discovering microdomains of signaling cells and studying the spatial spread of local input molecules, such as cytokines and growth factors (Oyler-Yaniv et al., 2017).

Microfluidic cell coculture systems enable controlled local signaling by compartmentalizing sender and receiver cells in closed environments with integrated separation valves. Depending on the sensitivity of receiver cells to global stimuli, sender cells can be either separated from receiver cells (Frank and Tay, 2015; Son et al., 2022; Watson et al., 2022) or confined together with receiver cells in the same chambers (Manfrin et al., 2019; Yang et al., 2022). However, achieving a 2D signaling model in a microfluidic system remains a challenge when global stimuli can also activate receiver cells. The geometric structure required for a 2D signal flow is difficult to achieve when sender and receiver cells are isolated with separation valves. The advantages and limitations of microfluidic systems as global input generators are also applicable to their applications in local signal generators. Native stimuli molecules with known concentrations allow for easy quantification of various types of global input. However, constructing a local signaling model with suspension cells still poses challenges.

The photodeprotection of caged input molecules have also been applied in local signal generation (Mancini et al., 2015). Sender cells can be activated through light irradiation of caged global stimuli. Another photoactivation-based method utilizes optogenetic tools to engineer photosensitive gene promotors in sender cells (Toettcher et al., 2013). Although both approaches are based on light irradiation, photodeprotection of caged stimuli is typically initiated by UV light, while photosensitive elements responsive to longer wavelengths of light can be used in optogenetic designs. The latter addresses the issue of phototoxicity associated with prolonged irradiation. This allows sender cells to continuously propagate local input signals. However, both methods have limitations, such as the restricted availability of photocaged stimuli and photo-responsive promotors.

Artificial cells have gained significant attention as substitutes for living cells (Xu et al., 2016; Buddingh and van Hest, 2017). These cell mimics provide a simplified platform for constructing cellular communities and storing and releasing interesting molecules with high precision. Artificial cells with biocompatibility have been employed as local signal senders (Toparlak et al., 2020). One key advantage is the high controllability of local signal generation. Signals can be released through passive diffusion, chemical induction (Toparlak et al., 2020) or light irradiation (Yang et al., 2020). Chemical induction allows for the generation of local input signals without directly stimulating living receiver cells. Additionally, the phototoxicity associated with light irradiation does not affect artificial cell senders. However, quantifying the released input molecules from artificial cells remains a challenge. One potential solution is to use fluorescently labeled input molecules. While studies on local signaling using artificial cells have been relatively limited to date, we believe that the unique advantages of artificial cells will facilitate further research in this area.

## Conclusions and future prospects

Global and local signal generators have significantly enhanced our understanding of temporal and spatial cellular signaling activities and cellular behaviors. In particular, microfluidic systems have emerged as powerful tools for investigating temporal activity of signaling pathways in single cells (Junkin et al., 2016; Sinha et al., 2022; Yang et al., 2022) and cell populations (Tay et al., 2010; Song et al., 2018; Mokashi et al., 2019). These systems allow precise delivery of various global inputs, such as pulse (Blazek et al., 2015; Ryu et al., 2015), continuous (Dettinger et al., 2018), and step-wise ramping (Song et al., 2018; Son et al., 2021), and ramping analog inputs (Mokashi et al., 2019), resulting in distinct cellular responses. Through the study of temporal behavior in individual cells, we have gained insights into fundamental biological processes like cell proliferation and differentiation (Zhu and Thompson, 2019), and immune response (Brubaker et al., 2015). For instance, perturbation of ERK activity with pulsatile inputs of EGF/NGF reveal that transient/sustained ERK dynamics induce proliferation/differentiation in PC-12 cells (Ryu et al., 2015). Continuous and pulse TNFα inputs were shown to elicit digital activation but analogue information processing of NF-κB in fibroblasts (Tay et al., 2010). Additionally, the delivery of linear/exponential stepwise ramping inputs of TNFα to fibroblasts demonstrates that NF-κB activity responds to the absolute difference in cytokine concentration rather than the concentration itself (Son et al., 2021). These findings highlight the power of microfluidic approaches in addressing complex biological questions that are challenging to investigate using conventional experiments conducted in well plates, which often allow for treatments with only continuous inputs or very few pulses of input signals (Ashall et al., 2009; Zhang et al., 2017). Despite significant progress, there is still much to uncover regarding the underlying signaling mechanisms and their implications, considering the diverse range of global input signals encountered by cells during biological events. The remarkable controllability of microfluidic systems opens up possibilities for exploring additional global input types, such as sinusoidal and triangle signals, which may provide further insights into temporal signaling responses in future studies.

The development of photocaging and photodeprotection-based global input generators has been a subject of ongoing research for years. This emerging technology has aided our exploration in control of cellular signaling activation. Several small molecule agonists of TLRs conjugated with 2-nitrobenzyl groups have been applied to control the activation of immune signaling pathways (Ryu et al., 2014; Govan et al., 2015; Stutts and Esser-Kahn, 2015). By exploring the signaling of TLRs using photoactivated agonists, we can gain insights into inflammatory responses and the innate immune system’s recognition of non-self, potentially leading to advancements in vaccine design. Photochemical techniques have also been employed for macromolecule inputs such as growth factors and cytokines. Caged dendritic molecular glues (Mogaki et al., 2019) and polymers (Perdue et al., 2020), have been utilized to photo-protect these protein input molecules, enabling their controlled release and activation of downstream signaling pathways. This has opened up possibilities for using photolabile molecular glues or polymers as universal inhibitors to control protein input-triggered signaling activation. By combined these techniques with reporter cells and time-lapse imaging (Yang et al., 2022), we can extend their applications to study temporal signaling dynamics in future. Furthermore, light irradiation can be programmed to create various input profiles, including multiple-wave and continuous stimulation. For example, photolabile molecular glues or polymers can be modified to photo-protect EGF/NGF and TNFα, allowing controlled activation of the ERK and NF-κB pathways, respectively. By applying specific input profiles of light irradiation, we can investigate the temporal dynamics of ERK and NF-κB signaling pathways.

Living cells have been adapted to serve as local signal generators using various approaches, as discussed in this review. These local signal generators can be easily extended to explore other cell types and signaling pathways, offering versatility and flexibility in experimental design. While artificial cells have not been widely applied as local signal generators in observing signaling dynamics in living receiver cells, recent studies have demonstrated their potential in controlled signaling activation in neural and HEK293 cells (Toparlak et al., 2020). The utilization of artificial cells as local signal generators faces challenges in building photo-responsive promotors and gene expression systems within these synthetic constructs. However, alternative strategies can be explored. For example, light-controlled DNA-mediated signaling between artificial cells has recently attracted attention (Yang et al., 2020). These artificial cells with adjustable permeability can store and release different DNA molecules conjugated with photolabile linkers under light irradiation. It raises the question of whether proteins, such as cytokine or growth factors, modified with photolabile linkers, can also be stored in artificial cells and released upon light irradiation.

In conclusion, the development of robust platforms for both global and local signal generation holds significant promise in enhancing our understanding of how cells encode and decode diverse input information across spatial and temporal dimensions. The impact of these signal generators is evident in their potential to elucidate the underlying signaling mechanisms governing temporal and spatial signaling dynamics, as well as cellular behaviors. We firmly believe that advancing and expanding upon the techniques discussed in this review will further propel the discovery of novel and intriguing signaling mechanisms.

## References

[B1] Ashall L. Horton C. A. Nelson D. E. Paszek P. Harper C. V. Sillitoe K. (2009). Pulsatile stimulation determines timing and specificity of NF-kappa B-dependent transcription. Science 324, 242–246. 10.1126/science.1164860 19359585 PMC2785900

[B2] Aufinger L. Simmel F. C. (2019). Establishing communication between artificial cells. Chem-Eur J. 25, 12659–12670. 10.1002/chem.201901726 31241792

[B3] Blazek M. Betz C. Hall M. N. Reth M. Zengerle R. Meier M. (2013). Proximity ligation assay for high-content profiling of cell signaling pathways on a microfluidic chip. Mol. Cell Proteomics 12, 3898–3907. 10.1074/mcp.M113.032821 24072685 PMC3861732

[B4] Blazek M. Santisteban T. S. Zengerle R. Meier M. (2015). Analysis of fast protein phosphorylation kinetics in single cells on a microfluidic chip. Lab. Chip 15, 726–734. 10.1039/c4lc00797b 25428717

[B5] Blum Y. Mikelson J. Dobrzynski M. Ryu H. Jacques M. A. Jeon N. L. (2019). Temporal perturbation of ERK dynamics reveals network architecture of FGF2/MAPK signaling. Mol. Syst. Biol. 15, e8947. 10.15252/msb.20198947 31777174 PMC6864398

[B91] Brisse M. Ly H. (2019). Comparative structure and function analysis of the RIG-I-like receptors: RIG-I and MDA5. Front. Immunol. 10. 10.3389/fimmu.2019.01586 PMC6652118 31379819

[B6] Brower K. Puccinelli R. R. Markin C. J. Shimko T. C. Longwell S. A. Cruz B. (2018). An open-source, programmable pneumatic setup for operation and automated control of single- and multi-layer microfluidic devices. Hardwarex 3, 117–134. 10.1016/j.ohx.2017.10.001 30221210 PMC6136661

[B7] Brubaker S. W. Bonham K. S. Zanoni I. Kagan J. C. (2015). Innate immune pattern recognition: A cell biological perspective. Annu. Rev. Immunol. 33, 257–290. 10.1146/annurev-immunol-032414-112240 25581309 PMC5146691

[B8] Buddingh B. C. van Hest J. C. M. (2017). Artificial cells: synthetic compartments with life-like functionality and adaptivity. Accounts Chem. Res. 50, 769–777. 10.1021/acs.accounts.6b00512 PMC5397886 28094501

[B9] Chen H. Y. Yu Z. H. Bai S. W. Lu H. X. Xu D. Chen C. (2019). Microfluidic models of physiological or pathological flow shear stress for cell biology, disease modeling and drug development. Trac-Trend Anal. Chem. 117, 186–199. 10.1016/j.trac.2019.06.023

[B10] Dekoninck S. Blanpain C. (2019). Stem cell dynamics, migration and plasticity during wound healing. Nat. Cell Biol. 21, 18–24. 10.1038/s41556-018-0237-6 30602767 PMC7615151

[B11] Dettinger P. Frank T. Etzrodt M. Ahmed N. Reimann A. Trenzinger C. (2018). Automated microfluidic system for dynamic stimulation and tracking of single cells. Anal. Chem. 90, 10695–10700. 10.1021/acs.analchem.8b00312 30059208

[B12] Devary Y. Rosette C. Di Donato J. A. Karin M. (1993). NF-κB activation by ultraviolet light not dependent on a nuclear signal. Science 261, 1442–1445. 10.1126/science.8367725 8367725

[B13] Discher D. E. Janmey P. Wang Y. L. (2005). Tissue cells feel and respond to the stiffness of their substrate. Science 310, 1139–1143. 10.1126/science.1116995 16293750

[B14] Dorrington M. G. Fraser I. D. C. (2019). NF-Kappa B signaling in macrophages: dynamics, crosstalk, and signal integration. Front. Immunol. 10, 705. 10.3389/fimmu.2019.00705 31024544 PMC6465568

[B15] Elani Y. Trantidou T. Wylie D. Dekker L. Polizzi K. Law R. V. (2018). Constructing vesicle-based artificial cells with embedded living cells as organelle-like modules. Sci. Rep-Uk 8, 4564. 10.1038/s41598-018-22263-3 PMC5852042 29540757

[B16] Fang G. C. Lu H. X. Es H. A. Wang D. J. Liu Y. Warkiani M. E. (2021). Unidirectional intercellular communication on a microfluidic chip. Biosens. Bioelectron. 175, 112833. 10.1016/j.bios.2020.112833 33288428

[B17] Farahani P. E. Reed E. H. Underhill E. J. Aoki K. Toettcher J. E. (2021). Signaling, deconstructed: using optogenetics to dissect and direct information flow in biological systems. Annu. Rev. Biomed. Eng. 23, 61–87. 10.1146/annurev-bioeng-083120-111648 33722063 PMC10436267

[B18] Frank T. Tay S. (2015). Automated co-culture system for spatiotemporal analysis of cell-to-cell communication. Lab. Chip 15, 2192–2200. 10.1039/c5lc00182j 25892510

[B19] Gao D. Liu H. X. Jiang Y. Y. Lin J. M. Liu H. (2012). Recent developments in microfluidic devices for *in vitro* cell culture for cell-biology research. Trac-Trend Anal. Chem. 35, 150–164. 10.1016/j.trac.2012.02.008

[B20] Govan J. M. Young D. D. Lively M. O. Deiters A. (2015). Optically triggered immune response through photocaged oligonucleotides. Tetrahedron Lett. 56, 3639–3642. 10.1016/j.tetlet.2015.01.165 26034339 PMC4448726

[B21] Hongdusit A. Liechty E. T. Fox J. M. (2020). Optogenetic interrogation and control of cell signaling. Curr. Opin. Biotech. 66, 195–206. 10.1016/j.copbio.2020.07.007 33053496

[B22] Hu H. G. Chen P. G. Wang G. Y. Wu J. J. Zhang B. D. Li W. H. (2020). Regulation of immune activation by optical control of TLR1/2 heterodimerization. Chembiochem 21, 1150–1154. 10.1002/cbic.201900591 31702879

[B23] Irimia D. (2010). Microfluidic technologies for temporal perturbations of chemotaxis. Annu. Rev. Biomed. Eng. 12, 259–284. 10.1146/annurev-bioeng-070909-105241 20450351 PMC3153854

[B24] Joesaar A. Yang S. Bogels B. van der Linden A. Pieters P. Kumar B. V. V. S. P. (2019). DNA-based communication in populations of synthetic protocells. Nat. Nanotechnol. 14, 369–378. 10.1038/s41565-019-0399-9 30833694 PMC6451639

[B25] Junkin M. Kaestli A. J. Cheng Z. Jordi C. Albayrak C. Hoffmann A. (2016). High-content quantification of single-cell immune dynamics. Cell Rep. 15, 411–422. 10.1016/j.celrep.2016.03.033 27050527 PMC4835544

[B26] Karoui H. Patwal P. S. Kumar B. V. V. S. P. Martin N. (2022). Chemical communication in artificial cells: basic concepts, design and challenges. Front. Mol. Biosci. 9, 880525. 10.3389/fmolb.2022.880525 35720123 PMC9199989

[B27] Kehl F. Cretu V. F. Willis P. A. (2021). Open-source lab hardware: A versatile microfluidic control and sensor platform. Hardwarex 10, e00229. 10.1016/j.ohx.2021.e00229 35607658 PMC9123481

[B28] Keifer O. Kambara K. Lau A. Makinson S. Bertrand D. (2020). Chemogenetics a robust approach to pharmacology and gene therapy. Biochem. Pharmacol. 175, 113889. 10.1016/j.bcp.2020.113889 32119836

[B29] Kellogg R. A. Tian C. Z. Lipniacki T. Quake S. R. Tay S. (2015). Digital signaling decouples activation probability and population heterogeneity. Elife 4, e08931. 10.7554/eLife.08931 26488364 PMC4608393

[B30] Kim D. Wu X. J. Young A. T. Haynes C. L. (2014). Microfluidics-based *in vivo* mimetic systems for the study of cellular biology. Accounts Chem. Res. 47, 1165–1173. 10.1021/ar4002608 PMC3993883 24555566

[B31] Klan P. Solomek T. Bochet C. G. Blanc A. Givens R. Rubina M. (2013). Photoremovable protecting groups in chemistry and biology: reaction mechanisms and efficacy. Chem. Rev. 113, 119–191. 10.1021/cr300177k 23256727 PMC3557858

[B32] Kramer M. M. Lataster L. Weber W. Radziwill G. (2021). Optogenetic approaches for the spatiotemporal control of signal transduction pathways. Int. J. Mol. Sci. 22, 5300. 10.3390/ijms22105300 34069904 PMC8157557

[B33] Lavoie H. Gagnon J. Therrien M. (2020). ERK signalling: A master regulator of cell behaviour, life and fate. Nat. Rev. Mol. Cell Bio 21, 607–632. 10.1038/s41580-020-0255-7 32576977

[B34] Leof E. B. (2000). Growth factor receptor signalling: location, location, location. Trends Cell Biol. 10, 343–348. 10.1016/S0962-8924(00)01795-5 10884687

[B35] Leopold A. V. Chernov K. G. Verkhusha V. V. (2018). Optogenetically controlled protein kinases for regulation of cellular signaling. Chem. Soc. Rev. 47, 2454–2484. 10.1039/c7cs00404d 29498733 PMC5882534

[B36] Li N. X. Karin M. (1998). Ionizing radiation and short wavelength UV activate NF-kappa B through two distinct mechanisms. P Natl. Acad. Sci. U. S. A. 95, 13012–13017. 10.1073/pnas.95.22.13012 PMC23690 9789032

[B37] Li P. L. Markson J. S. Wang S. Chen S. H. Vachharajani V. Elowitz M. B. (2018). Morphogen gradient reconstitution reveals Hedgehog pathway design principles. Science 360, 543–548. 10.1126/science.aao0645 29622726 PMC6516753

[B38] Mancini R. J. Stutts L. Moore T. Esser-Kahn A. P. (2015). Controlling the origins of inflammation with a photoactive lipopeptide immunopotentiator. Angew. Chem. Int. Ed. 54, 5962–5965. 10.1002/anie.201500416 PMC4539149 25800006

[B39] Manfrin A. Tabata Y. Paquet E. R. Vuaridel A. R. Rivest F. R. Naef F. (2019). Engineered signaling centers for the spatially controlled patterning of human pluripotent stem cells. Nat. Methods 16 640-648. 10.1038/s41592-019-0455-2 31249412

[B40] Moenter S. M. Brand R. M. Midgley A. R. Karsch F. J. (1992). Dynamics of gonadotropin-releasing-hormone release during a pulse. Endocrinology 130 503–510. 10.1210/endo.130.1.1727719 1727719

[B41] Mogaki R. Okuro K. Ueki R. Sando S. Aida T. (2019). Molecular glue that spatiotemporally turns on protein-protein interactions. J. Am. Chem. Soc. 141, 8035–8040. 10.1021/jacs.9b02427 30977371

[B42] Mokashi C. S. Schipper D. L. Qasaimeh M. A. Lee R. E. C. (2019). A system for analog control of cell culture dynamics to reveal capabilities of signaling networks. Iscience 19, 586–596. 10.1016/j.isci.2019.08.010 31446223 PMC6713801

[B43] Mrksich M. (2000). A surface chemistry approach to studying cell adhesion. Chem. Soc. Rev. 29, 267–273. 10.1039/a705397e

[B44] Mudla A. Jiang Y. F. Arimoto K. I. Xu B. X. Rajesh A. Ryan A. P. (2020). Cell-cycle-gated feedback control mediates desensitization to interferon stimulation. Elife 9, e58825. 10.7554/eLife.58825 32945770 PMC7500952

[B45] Mukwaya V. Mann S. Dou H. J. (2021). Chemical communication at the synthetic cell/living cell interface. Commun. Chem. 4, 161. 10.1038/s42004-021-00597-w 36697795 PMC9814394

[B46] Niederholtmeyer H. Chaggan C. Devaraj N. K. (2018). Communication and quorum sensing in non-living mimics of eukaryotic cells. Nat. Commun. 9, 5027. 10.1038/s41467-018-07473-7 30487584 PMC6261949

[B47] O'Dea E. L. Kearns J. D. Hoffmann A. (2008). UV as an amplifier rather than inducer of NF-kappa B activity. Mol. Cell 30, 632–641. 10.1016/j.molcel.2008.03.017 18538661 PMC2682114

[B93] Onomoto K. Onoguchi K. Yoneyama M. (2021). Regulation of RIG-I-like receptor-mediated signaling: interaction between host and viral factors. Cell Mol. Immunol. 18, 539–555. 10.1038/s41423-020-00602-7 33462384 PMC7812568

[B48] O'Shea J. J. Murray P. J. (2008). Cytokine signaling modules in inflammatory responses. Immunity 28, 477–487. 10.1016/j.immuni.2008.03.002 18400190 PMC2782488

[B49] Osborn O. Olefsky J. M. (2012). The cellular and signaling networks linking the immune system and metabolism in disease. Nat. Med. 18, 363–374. 10.1038/nm.2627 22395709

[B50] Oyler-Yaniv A. Oyler-Yaniv J. Whitlock B. M. Liu Z. D. Germain R. N. Huse M. (2017). A tunable diffusion-consumption mechanism of cytokine propagation enables plasticity in cell-to-cell communication in the immune system. Immunity 46, 609–620. 10.1016/j.immuni.2017.03.011 28389069 PMC5442880

[B51] Perdue L. A. Do P. David C. Chyong A. Kellner A. V. Ruggieri A. (2020). Optical control of cytokine signaling via bioinspired, polymer-induced latency. Biomacromolecules 21, 2635–2644. 10.1021/acs.biomac.0c00264 32374589 PMC8496955

[B52] Piehler A. Ghorashian N. Zhang C. Tay S. (2017). Universal signal generator for dynamic cell stimulation. Lab. Chip 17, 2218–2224. 10.1039/C7LC00531H 28573304 PMC5767101

[B53] Purvis J. E. Lahav G. (2013). Encoding and decoding cellular information through signaling dynamics. Cell 152, 945–956. 10.1016/j.cell.2013.02.005 23452846 PMC3707615

[B54] Raper J. Galvan A. (2022). Applications of chemogenetics in non-human primates. Curr. Opin. Pharmacol. 64, 102204. 10.1016/j.coph.2022.102204 35307295 PMC9167257

[B92] Rehwinkel J. Gack M. U. (2020). RIG-I-like receptors: their regulation and roles in RNA sensing. Nat. Rev. Immunol. 20, 537–551. 10.1038/s41577-020-0288-3 32203325 PMC7094958

[B55] Rho H. S. Yang Y. Hanke A. T. Ottens M. Terstappen L. W. M. M. Gardeniers H. (2016). Programmable v-type valve for cell and particle manipulation in microfluidic devices. Lab. Chip 16, 305–311. 10.1039/c5lc01206f 26648416

[B90] Rojas V. Larrondo L. F. (2022). Coupling cell communication and optogenetics: implementation of a light-inducible intercellular system in yeast. ACS Synth. Biol. 10.1021/acssynbio.2c00338 PMC9872819 36534043

[B56] Ryu H. Chung M. Dobrzynski M. Fey D. Blum Y. Lee S. S. (2015). Frequency modulation of ERK activation dynamics rewires cell fate. Mol. Syst. Biol. 11, 838. 10.15252/msb.20156458 26613961 PMC4670727

[B57] Ryu K. A. McGonnigal B. Moore T. Kargupta T. Mancini R. J. Esser-Kahn A. P. (2017). Light guided *in-vivo* activation of innate immune cells with photocaged TLR 2/6 agonist. Sci. Rep-Uk 7, 8074. 10.1038/s41598-017-08520-x PMC5556111 28808328

[B58] Ryu K. A. Stutts L. Tom J. K. Mancini R. J. Esser-Kahn A. P. (2014). Stimulation of innate immune cells by light-activated TLR7/8 agonists. J. Am. Chem. Soc. 136, 10823–10825. 10.1021/ja412314j 25029205 PMC4132957

[B59] Sanchez E. Huse M. (2018). Spatial and temporal control of T cell activation using a photoactivatable agonist. Jove-J Vis. Exp., 56655. 10.3791/56655 PMC6100773 29757266

[B60] Serafini N. Vosshenrich C. A. J. Di Santo J. P. (2015). Transcriptional regulation of innate lymphoid cell fate. Nat. Rev. Immunol. 15, 415–428. 10.1038/nri3855 26065585

[B61] Seth R. B. Sun L. J. Chen Z. J. J. (2006). Antiviral innate immunity pathways. Cell Res. 16, 141–147. 10.1038/sj.cr.7310019 16474426

[B62] Sia S. K. Whitesides G. M. (2003). Microfluidic devices fabricated in poly(dimethylsiloxane) for biological studies. Electrophoresis 24, 3563–3576. 10.1002/elps.200305584 14613181

[B63] Sinha N. Subedi N. Tel J. (2018). Integrating immunology and microfluidics for single immune cell analysis. Front. Immunol. 9, 2373. 10.3389/fimmu.2018.02373 30459757 PMC6232771

[B64] Sinha N. Yang H. Janse D. Hendriks L. Rand U. Hauser H. (2022). Microfluidic chip for precise trapping of single cells and temporal analysis of signaling dynamics. Commun. Eng. 1, 18. 10.1038/s44172-022-00019-2

[B65] Son M. Frank T. Holst-Hansen T. Wang A. G. Junkin M. Kashaf S. S. (2022). Spatiotemporal NF-kappa B dynamics encodes the position, amplitude, and duration of local immune inputs. Sci. Adv. 8, eabn6240. 10.1126/sciadv.abn6240 36044569 PMC9432835

[B66] Son M. J. Wang A. G. Tu H. L. Metzig M. O. Patel P. Husain K. (2021). NF-kappa B responds to absolute differences in cytokine concentrations. Sci. Signal 14, eaaz4382. 10.1126/scisignal.aaz4382 34211635 PMC8244746

[B67] Song J. Ryu H. Chung M. Kim Y. Blum Y. Lee S. S. (2018). Microfluidic platform for single cell analysis under dynamic spatial and temporal stimulation. Biosens. Bioelectron. 104, 58–64. 10.1016/j.bios.2017.12.038 29306762

[B68] Spiller D. G. Wood C. D. Rand D. A. White M. R. H. (2010). Measurement of single-cell dynamics. Nature 465, 736–745. 10.1038/nature09232 20535203

[B69] Stutts L. Esser-Kahn A. P. (2015). A light-controlled TLR4 agonist and selectable activation of cell subpopulations. Chembiochem 16, 1744–1748. 10.1002/cbic.201500164 26097006 PMC4881745

[B70] Sumit M. Takayama S. Linderman J. J. (2017). New insights into mammalian signaling pathways using microfluidic pulsatile inputs and mathematical modeling. Integr. Biol-Uk 9, 6–21. 10.1039/c6ib00178e PMC5259548 27868126

[B71] Sun Y. Liu W. Z. Liu T. Feng X. Yang N. Zhou H. F. (2015). Signaling pathway of MAPK/ERK in cell proliferation, differentiation, migration, senescence and apoptosis. J. Recept Sig Transd 35, 600–604. 10.3109/10799893.2015.1030412 26096166

[B72] Tay S. Hughey J. J. Lee T. K. Lipniacki T. Quake S. R. Covert M. W. (2010). Single-cell NF-kappa B dynamics reveal digital activation and analogue information processing. Nature 466, 267–271. 10.1038/nature09145 20581820 PMC3105528

[B73] Thorsen T. Maerkl S. J. Quake S. R. (2002). Microfluidic large-scale integration. Science 298, 580–584. 10.1126/science.1076996 12351675

[B74] Tischer D. Weiner O. D. (2014). Illuminating cell signalling with optogenetic tools. Nat. Rev. Mol. Cell Bio 15, 551–558. 10.1038/nrm3837 25027655 PMC4145075

[B75] Toettcher J. E. Weiner O. D. Lim W. A. (2013). Using optogenetics to interrogate the dynamic control of signal transmission by the ras/erk module. Cell 155, 1422–1434. 10.1016/j.cell.2013.11.004 24315106 PMC3925772

[B76] Toparlak O. D. Zasso J. Bridi S. Dalla Serra M. Macchi P. Conti L. (2020). Artificial cells drive neural differentiation. Sci. Adv. 6, eabb4920. 10.1126/sciadv.abb4920 32948587 PMC7500934

[B77] Tsai Y. H. Doura T. Kiyonaka S. (2021). Tethering-based chemogenetic approaches for the modulation of protein function in live cells. Chem. Soc. Rev. 50, 7909–7923. 10.1039/d1cs00059d 34114579

[B78] Villarino A. V. Kanno Y. O'Shea J. J. (2017). Mechanisms and consequences of Jak-STAT signaling in the immune system. Nat. Immunol. 18, 374–384. 10.1038/ni.3691 28323260 PMC11565648

[B79] Watson C. Liu C. Ansari A. Miranda H. C. Somoza R. A. Senyo S. E. (2022). Multiplexed microfluidic chip for cell co-culture. Analyst 147, 5409–5418. 10.1039/d2an01344d 36300548 PMC10077866

[B80] Watson C. Senyo S. (2019). All-in-one automated microfluidics control system. Hardwarex 5, e00063. 10.1016/j.ohx.2019.e00063 31192312 PMC6561480

[B81] Wegener M. Hansen M. J. Driessen A. J. M. Szymanski W. Feringa B. (2017). Photocontrol of antibacterial activity: shifting from UV to red light activation. J. Am. Chem. Soc. 139, 17979–17986. 10.1021/jacs.7b09281 29136373 PMC5730949

[B82] White J. A. Streets A. M. (2018). Controller for microfluidic large-scale integration. Hardwarex 3, 135–145. 10.1016/j.ohx.2017.10.002 30775638 PMC6373447

[B83] Xu C. Hu S. Chen X. Y. (2016). Artificial cells: from basic science to applications. Mater Today 19, 516–532. 10.1016/j.mattod.2016.02.020 PMC5222523 28077925

[B84] Yang H. W. Sinha N. Rand U. Hauser H. Koster M. de Greef T. F. A. (2022). A universal microfluidic approach for integrated analysis of temporal homocellular and heterocellular signaling and migration dynamics. Biosens. Bioelectron. 211, 114353. 10.1016/j.bios.2022.114353 35594624

[B85] Yang S. Pieters P. A. Joesaar A. Bogels B. W. A. Brouwers R. Myrgorodska I. (2020). Light-activated signaling in DNA-encoded sender-receiver architectures. Acs Nano 14, 15992–16002. 10.1021/acsnano.0c07537 33078948 PMC7690052

[B86] Yuan Y. Jiang Y. C. Sun C. K. Chen Q. M. (2016). Role of the tumor microenvironment in tumor progression and the clinical applications (Review). Oncol. Rep. 35, 2499–2515. 10.3892/or.2016.4660 26986034

[B87] Zhang K. Cu B. X. (2015). Optogenetic control of intracellular signaling pathways. Trends Biotechnol. 33, 92–100. 10.1016/j.tibtech.2014.11.007 25529484 PMC4308517

[B88] Zhang Q. H. Gupta S. Schipper D. L. Kowalczyk G. J. Mancini A. E. Faeder J. R. (2017). NF-Kappa B dynamics discriminate between TNF doses in single cells. Cell Syst. 5, 638–645. 10.1016/j.cels.2017.10.011 29128333 PMC5746429

[B89] Zhu J. J. Thompson C. B. (2019). Metabolic regulation of cell growth and proliferation. Nat. Rev. Mol. Cell Bio 20, 436–450. 10.1038/s41580-019-0123-5 30976106 PMC6592760

